# A rapid evaluation of the UK Health Security Agency’s New Variant Assessment Platform global genomic surveillance programme

**DOI:** 10.1371/journal.pgph.0005578

**Published:** 2025-12-05

**Authors:** Koren Sanderson, Nicola Love, Ameze Simbo, Aishwarya Krishna, Constantina Laou, Charlotte Gallagher, Leena Inamdar, Charles R. Beck

**Affiliations:** 1 Evaluation & Epidemiological Science Division, Chief Scientific Officer Group, UK Health Security Agency, Salisbury, England; 2 Formerly New Variant Assessment Platform, Chief Scientific Officer Group, UK Health Security Agency, London, England; PLOS: Public Library of Science, UNITED STATES OF AMERICA

## Abstract

The New Variant Assessment Platform (NVAP) was a global genomic surveillance programme established by the UK Health Security Agency (UKHSA) in April 2021. The NVAP offered sequencing and biological assessment capabilities, technical assistance, and training to support the detection and assessment of new variants of SARS-CoV-2 and other high-priority pathogens globally. We present findings from a rapid evaluation of the NVAP. A mixed-method evaluation was conducted between June and November 2023 using a framework published by the Organisation for Economic Co-operation and Development (OECD). A review of the NVAP documentation, online surveys with partners and programme informants, and structured interviews with strategic informants were undertaken. Survey data was analysed using descriptive statistics, and interview data and the NVAP documentation were analysed thematically. Survey responses were received from 31/46 partners (67%) and structured interviews were conducted with 11/13 strategic stakeholders (85%). Partners provided evidence that the programme had met its overarching objectives. The NVAP enabled partners to start undertaking genomic surveillance or strengthened their existing capabilities. The importance of maintaining the global genomic surveillance capabilities developed during the COVID-19 pandemic and promoting ongoing programme sustainability in an evolving global genomic surveillance landscape was a recurring theme. Further themes included developing a pathogen agnostic approach, strengthening collaborations, promoting data sharing, and enhancing aspects of the NVAP offer. The NVAP achieved its overarching objectives, and qualitative findings showed it was responsive to stakeholder needs. The programme closed on 31 March 2024 following public health management of COVID-19 aligning to other common respiratory illnesses. We recommend that global health stakeholders should build resilient and adaptive surveillance systems, ensuring rapid detection and response to future health threats. Future research should focus on the longer-term utility and sustainability of global genomic surveillance initiatives and programmes.

## Introduction

Throughout the COVID-19 pandemic, genomic surveillance was fundamental in informing measures to reduce the impact of SARS-CoV-2 variants globally [1]. The importance of supporting low-and middle-income countries (LMICs) to strengthen SARS-CoV-2 genomic surveillance capacity locally and provide sustainable funding for sequencing capacity and outbreak analytics was recognised as a key area for pandemic response [1]. However, many LMICs faced logistical, technological, and financial barriers in performing genomic sequencing at the required scale [2]. Countries with large-scale sequencing capacity and extensive specialist expertise such as the United Kingdom (UK) were uniquely positioned to work collaboratively to address global genomic sequencing capability gaps [3].

The New Variant Assessment Platform (NVAP) was a global genomic surveillance programme led by the UK Health Security Agency (UKHSA; formerly Public Health England), with a cross-government board providing strategic oversight and direction [3]. The programme was established in April 2021 and offered sequencing and biological assessment capabilities, technical assistance, and training to support the detection and assessment of new variants of SARS-CoV-2 and other high-priority pathogens globally, with the aim of strengthening national and global health security [4]. Through the NVAP, UKHSA worked with global partners to strengthen localised genomic sequencing capabilities and improve global surveillance via a model tailored to the needs of individual countries or regions [3]. The NVAP had five overarching objectives (NVAP O1-O5) relating to capability strengthening, capacity strengthening, surge capacity, risk assessment and global system strengthening (Table 1). As of July 2023, the NVAP provided targeted support to nine bilateral partners and seven regional level partners in collaboration with the World Health Organization (WHO). The programme had been funded as part of the UKHSA’s COVID-19 response since April 2021.

**Table 1:** The NVAP Objectives.

Objective	Objective Description
**NVAP O1**	Provide technical assistance, training, and mentorship to build sequencing capability within partner institutions.
**NVAP O2**	Support countries to increase their pathogen sequencing capacity by procurement of reagents and equipment.
**NVAP O3**	Offer surge sequencing capacity within UKHSA labs if requested by countries with limited or no in-country capacity.
**NVAP O4**	Provide a pathway for biological risk assessment for pathogens of concern requiring specialist biological containment facilities through UKHSA.
**NVAP O5**	Strengthen global genomic surveillance, global leadership and advocacy, and global health security and pandemic preparedness initiatives.

In May 2023, the World Health Organization (WHO) declared that COVID-19 no longer fitted the definition of a Public Health Emergency of International Concern (PHEIC) [5]. It was important to understand the role of the NVAP in an evolving public health landscape, and to ensure that lessons related to genomic surveillance were learnt from the COVID-19 pandemic response. Understanding the programme’s successes and learnings is fundamental to inform the future development and implementation of effective global genomic surveillance programmes. In this context, a rapid evaluation was undertaken to assess the activities of the NVAP against the programme aim and objectives and understand the views and experiences of selected stakeholders involved in the implementation and delivery of the programme. The evaluation findings have been discussed and recommendations proposed in context of strategic priorities for international pathogen genomic surveillance.

## Materials and methods

The evaluation was conducted by UKHSA’s Evaluation and Epidemiological Science Division. To maintain independence, the NVAP team were not involved in the evaluation analysis process, interpretation of findings or generating recommendations.

### Evaluation framework

The NVAP was evaluated using the Organisation for Economic Co-operation and Development (OECD) Development Assistance Committee (DAC) evaluation framework, which includes criteria appropriate for evaluating complex global programmes [6]. The framework was used to assess the relevance, coherence, effectiveness, efficiency, impact, and sustainability of the NVAP. While all domains were assessed as part of the rapid evaluation, this manuscript specifically focuses on aspects of effectiveness, impact, and sustainability as these were the key themes which emerged.

### The NVAP stakeholders

Partners were selected for inclusion in the NVAP using prioritisation criteria agreed by the NVAP Programme Board, UK government stakeholders and international strategic partners. Three stakeholder groups were involved in developing and co-delivering the NVAP programme, defined for the purposes of this evaluation in Table 2.

**Table 2:** The NVAP Stakeholder Group Definitions, Survey Aims and Stakeholders Contacted.

Stakeholder Group	Stakeholder Definition	Stakeholder Survey Aim	Stakeholders Contacted
**Bilateral Partner**	A country or institution with a bilateral agreement with the NVAP, informing and receiving tailored support from the NVAP at the national level.	To capture experiences of the NVAP offer in each county (focusing on NVAP O1-O4), lessons learnt, programme strengths and limitations, and future expectations of the programme.	18 (10 organisations) *
**Regional Partner** **E.g., World Health Organization Regional Offices**	A regional or global organisation informing and receiving tailored support from the NVAP at the regional level.	To capture experiences of developing and delivering NVAP O1 and O2, lessons learnt, programme strengths and limitations, and future expectations of the programme.	11 (5 organisations) *
**Informant** **E.g., UKHSA**	UK and international partners involved in supporting the development and delivery of the overarching NVAP programme, for example by providing technical assistance and training.	To capture experiences of developing and delivering NVAP O1-O4, lessons learnt, programme strengths and limitations, and future expectations of the programme.	17 (8 organisations)

*For the purposes of the evaluation, one regional partner was sent the bilateral survey as it aligned more closely with the nature of their collaboration with the NVAP and is henceforth referred to as a bilateral partner.

At least one representative from all bilateral partner, regional partner, and informant organisations (57 individuals from 26 organisations) involved in the NVAP were invited to participate in this evaluation. Partners were asked to provide feedback via the relevant online survey (as defined in Table 2; see S1 Survey, S2 Survey and S3 Survey) or an interview on behalf of their organisation, depending on the nature of their involvement. Partners had up to five weeks to self-complete the online survey with targeted reminder emails sent to non-respondents, and interviews were arranged at a mutually convenient time.

### Data collection and analysis

**Fig 1:**
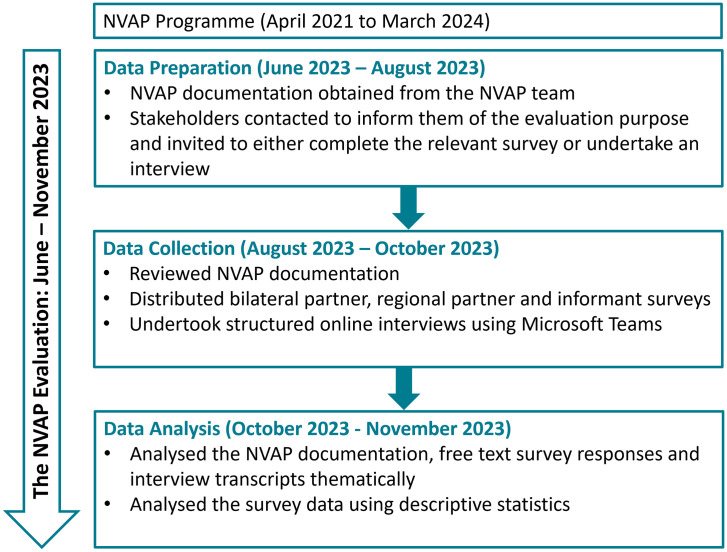
The NVAP Evaluation Data Collection and Analysis Process.

A mixed-method approach was used, with data collection performed by three investigators between August and October 2023, as outlined in Fig 1.

Programme documentation from April 2021 to July 2023 was provided by the NVAP team and reviewed and thematically analysed. Online surveys tailored to the three stakeholder groups were designed using SelectSurvey (an online survey software), to capture partners’ views and experiences of the NVAP as outlined in Table 2. Links to the surveys were shared with partners via email and were self-completed. Structured face-to-face online interviews with key programme informants captured views on NVAP O5. Interviews were recorded and transcribed using Microsoft Teams with transcripts checked for accuracy. Survey and interview responses were analysed using thematic analysis and descriptive statistics.

### Ethics statement

Advice was sought from the UKHSA research ethics and governance group (REGG). However, ethical approval for this type of study (service evaluation) is not required by UKHSA, which uses criteria based on guidance published by the UK Health Research Authority [7]. Study recruitment took place between 01/08/2023 and 31/10/2023. Evaluation participants were informed of the purpose of this evaluation, their right to confidentiality, and that any data would be handled and stored in compliance with the Data Protection Act (2018), guidelines established by the local Caldicott guardian and relevant UKHSA policies and procedures in advance of data collection. Prior written consent to take part in an interview was obtained via email and verbal consent was obtained at the beginning of each interview. Implied consent was deemed appropriate for the online survey through completion and submission of the online form.

## Results

Completed survey responses were received from 31/46 stakeholders (67% response rate); 10/18 bilateral partners (56% response rate), 8/11 regional partners (73% response rate), and 13/17 informants (76% response rate). Structured interviews were conducted with 11/13 strategic informants (85% response rate) from UK government and international organisations. Two of the interviewed stakeholders also provided a response to an NVAP evaluation survey. Approximately 100 programme documents were reviewed.

### Effectiveness: Did the NVAP achieve its objectives?

A key strength of the programme was that it employed a bottom-up approach, with the offer and objectives developed collaboratively with bilateral and regional partners to address partner needs. This included capability strengthening (NVAP O1) and prioritisation of in-country capacity building (NVAP O2) rather than reliance on UK genomic sequencing infrastructure for sample processing.

“*It’s not a top-down approach. It’s a bottom-up approach that they are really listening for, where can they [the NVAP] fit in?” (*Interview Participant)

**Fig 2:**
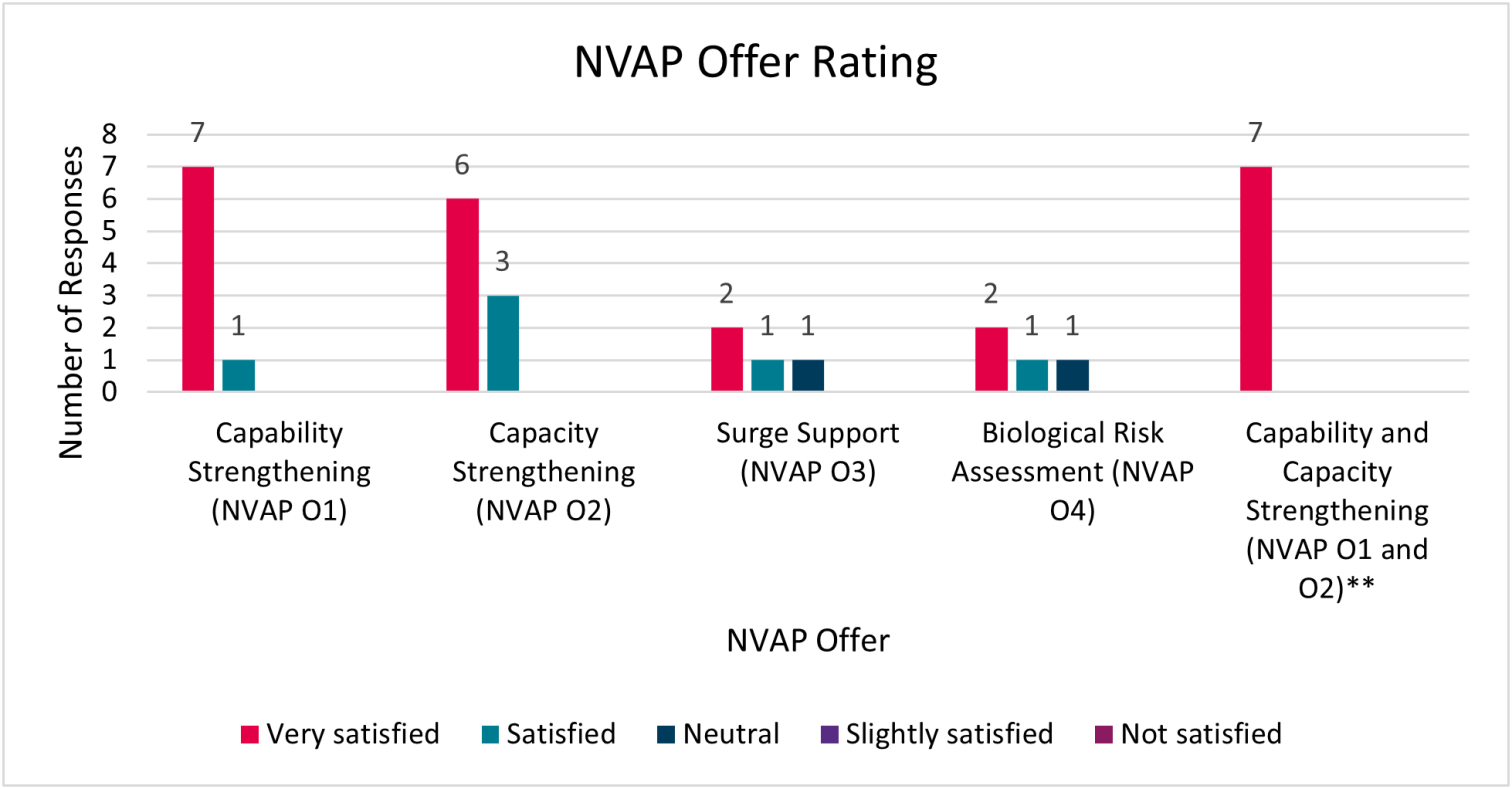
The NVAP Offer Rating. ** Regional partners were asked to rate the NVAP O1 and NVAP O2 offers together.

Capability strengthening activities (NVAP O1) focused on the delivery of training and the provision of technical assistance and was well-received by partners; all bilateral (n = 8) and regional (n = 7) partner respondents were ‘very satisfied’ or ‘satisfied’ by the offer (Fig 2).

Training needs were identified through a comprehensive questionnaire, with respondents highlighting the need for further wet lab sequencing, epidemiology, and bioinformatics training (NVAP documentation). Structured training courses were co-designed and co-delivered with the target audience, and the offer developed and rolled out in a phased manner. As of July 2023, participants from 57 institutions across 53 countries had attended training sessions delivered by the NVAP, with post-training course evaluations showing that the sessions were well regarded by partners (NVAP documentation). Formal training was supported by technical assistance including regular scientific technical exchanges, deployment of UKHSA advisors, and the development of technical guidance and protocols (NVAP documentation), which were “*adapt[ed] to regional requests as well as direct country requests*” (Regional Partner). The NVAP also promoted quality by sponsoring 18 laboratories to participate in a pilot External Quality Assessment (EQA) scheme and for 20 institutions across 17 countries to undertake NEQAS registration [8] (NVAP documentation).

“*Both online training and in-person training were systematic and excellent*” (Regional Partner)

To support development of the in-country delivery of genomic sequencing, a key emphasis of the NVAP was in-country capacity strengthening activities (NVAP O2) with this support rated positively by partners (All bilateral (n = 9) and regional (n = 7) partner respondents were ‘very satisfied’ or ‘satisfied’ by the offer; Fig 2). The support most frequently received by bilateral partners was the procurement of reagents (n = 8, 89%) and equipment (n = 8, 89%). Regional partners were most often supported with the scoping of local pathogen genomic surveillance capacity (n = 7, 88%) and strengthening of regional hubs (n = 6, 75%). Initial challenges with procurement and logistics, particularly because of UKHSA procurement processes, were recognised limitations of early programme delivery that were subsequently resolved.

“*The availability of consumables continues to be an important limitation of our work, so the donation of reagents by [the] NVAP gives peace of mind and continuity to our work*” (Bilateral Partner)

Surge sequencing support (NVAP O3) and biological risk assessment using UK infrastructure (NVAP O4) were also offered by the NVAP (Fig 2). Only three partner countries took up surge sequencing support, which was restricted to the acute phase of the pandemic (NVAP documentation). Interview participants acknowledged that there were logistical, technical, and political challenges to sending and sharing specimens globally, which limited uptake of this offer. Similarly, only four respondents reported receiving biological risk assessment support (virus isolation and phenotypic characterization).

While there were recognised challenges with sharing and sending biological specimens between countries, global genomic surveillance was strengthened through the facilitation of data sharing (NVAP O5), while ensuring the intellectual property of data generated belonged to partners, rather than the UK. Partners were encouraged to submit genomic data generated through the NVAP activities to the Global Initiative on Sharing All Influenza Data (GISAID) platform, which supports the rapid sharing of data from priority pathogens including SARS-CoV-2 [9]. Furthermore, the NVAP supported partners with data validation, cleaning, and interpretation to ensure that data uploaded to GISAID was high quality and timely.

*“One of the, the most unique things that [the] NVAP has been able to achieve, is the agreement with partners to do data sharing with us [UKHSA]”* (Interview Participant)

Through the NVAP activities, the UK was able to demonstrate strategic and technical leadership in global genomic strengthening activities (NVAP O5). The NVAP core team contributed to the International Pathogen Surveillance Network (IPSN), a global network to accelerate progress in pathogen genomics [10], the 100 Days Mission report [4] and supported development of the WHO global genomic surveillance strategy ([11,12], NVAP documentation). There was widespread praise for the NVAP team’s engagement with global partners. However, interview participants noted that the NVAP could demonstrate more global leadership and innovation going forward.

*“Over the last year in particular, [the] NVAP has become really quite influential and important in that programme [IPSN]” (Interview Participant)*

**Fig 3:**
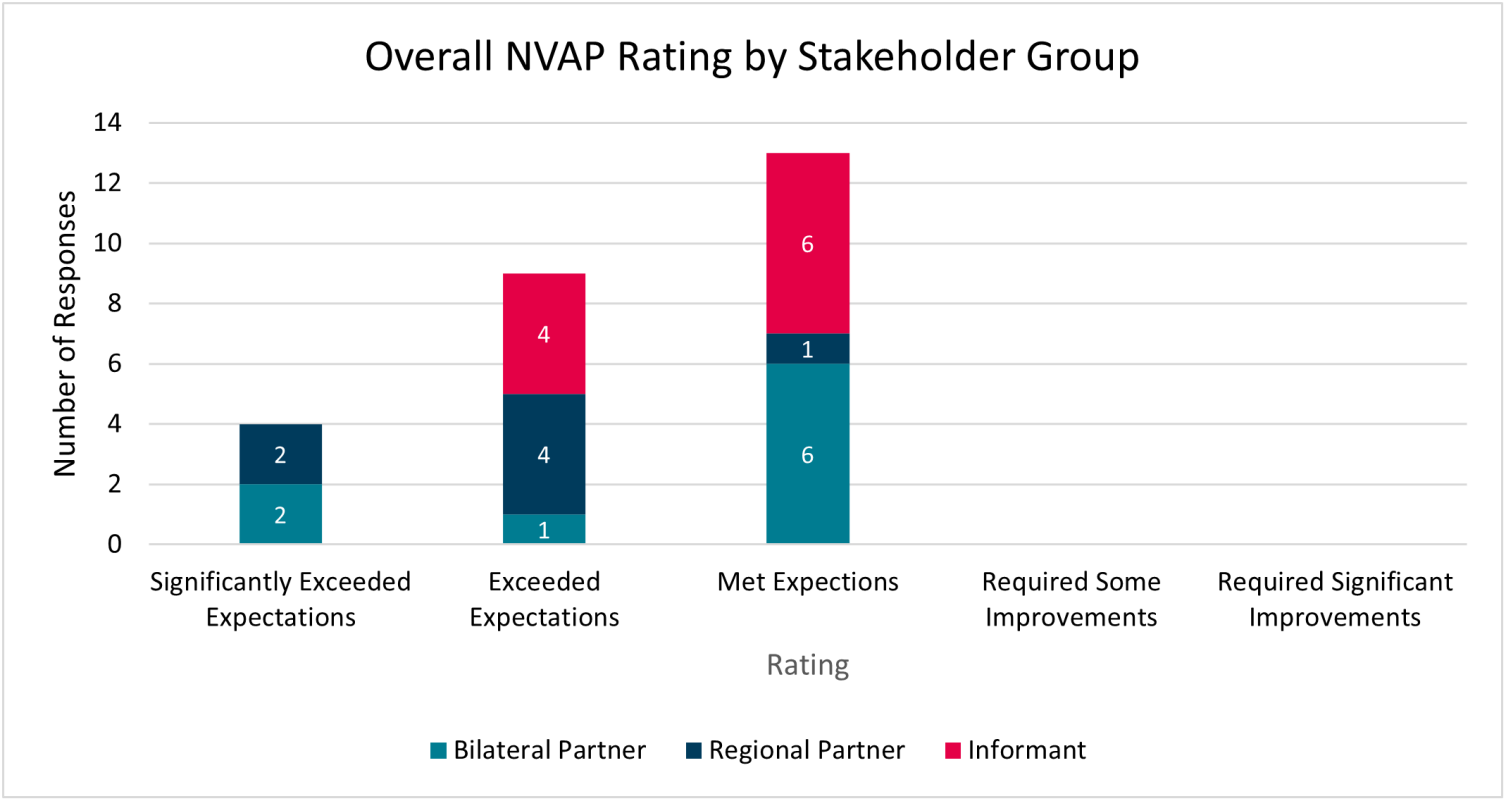
Overall The NVAP Rating by Stakeholder Group.

Overall, respondents providing an overall NVAP rating (26/31 questionnaire respondents) agreed that the implementation and delivery of the NVAP met (n = 13, 50%), exceeded (n = 9, 35%) or significantly exceeded (n = 4, 15%) their expectations (Fig 3). The most positive ratings were received from regional partners, with 6 out of 7 regional partner respondents (86%) reflecting that the support either exceeded or significantly exceeded their expectations. The support provided was “*all-encompassing*” (Bilateral Partner) and “*really made a difference*” (Regional Partner). Responders praised the collaborative, bottom-up approach taken with partners highlighting the positive relationships established with the NVAP team, and the expertise and technical leadership of the range of UK agencies and academic partners involved in the programme.

The NVAP theory of change model was developed to explain how the programme aimed to achieve its desired long-term outcomes and impacts, and outlined the programme inputs, activities, milestones, outputs and impacts of the NVAP. There was evidence to support this model, particularly the inputs, activities, outputs, and milestones. However, it will take time for the proposed outcomes and inputs of the programme to become fully embedded.

### Impact: Did the NVAP make a difference?

Partners reported that the NVAP support had enabled them to start undertaking genomic surveillance or strengthened their existing capacity and capability. The NVAP’s capacity strengthening support (NVAP O2) increased bilateral partners (n = 8) sequencing throughput, due to training and support (n = 7), provision of reagents and equipment (n = 6) and technical assistance (n = 6). This resulted in real-time SARS-CoV-2 surveillance, timely variant detection, and expanded surveillance scope and diagnostic capabilities (NVAP documentation).

*“[The] NVAP support to the region successfully aided the implementation of COVID-19 sequencing across the region”* (Regional Partner)

Reported benefits of the capability strengthening offer (NVAP O1) included increased technical proficiency, improved approaches to monitoring and controlling infectious diseases, and using genomic and epidemiological data effectively to inform public health decisions. The EQA support resulted in improved data quality, enhanced confidence in results, and increased credibility for the NVAP partners by aligning their diagnostic services with international quality standards.

Promoting global data sharing to inform risk assessments and public health action was consistently highlighted as a programme benefit, and the NVAP “*really raised awareness of the necessity of doing the sequencing but also more importantly, sharing the results of those sequences internationally*” (Interview Participant). Data uploaded to GISAID by the NVAP partners was accessible globally including by UKHSA’s variant horizon scanning system and used to support the UK’s domestic risk assessment. An informant noted that closely monitoring pathogens also allowed the NVAP’s procurement strategy to adapt to address potential threats.

The relationships and networks developed through the NVAP, particularly with bilateral partners, regional partners and WHO, increased the impact and efficiency of the programme. An interview participant noted that the NVAP built relationships with countries in a practical way, by offering valuable assistance during the COVID-19 pandemic.. The NVAP’s diplomatic impact was also recognised: “*I think there’s huge diplomatic value*” (Interview Participant).

It was observed that the NVAP’s contribution to global strategies and forums was leading to “*better pandemic preparedness, incident response, and utilisation of the genomic surveillance in country*” (Interview Participant) and the programme was able to advocate “*the importance of genomic surveillance and its utility in public health*” (Interview Participant). It was agreed that the NVAP support had resulted in improved preparation for future pandemic and epidemic threats through capability expansion.

### Sustainability: Will the benefits last?

The NVAP was setup rapidly as part of the UK’s COVID-19 response, and partners reflected that there had been less input into the longer-term sustainability of the programme. However, in the context of COVID-19 no longer being a PHEIC and the re-emergence of other pathogens, several partners highlighted the importance of considering programme sustainability.

*“It’s really important to secure the future and sustainability of the genomic surveillance work that is being led by UKHSA through [the] NVAP*” (Interview Participant)

Maintaining the genomic surveillance momentum developed during the COVID-19 pandemic, particularly following significant investment in the programme, was a key concern of stakeholders. The risks of expanding too quickly were acknowledged and included losing the focus of the programme and a stagnation or reversal of programme benefits, for example countries ceasing to provide genomic surveillance. It was suggested that the NVAP must proactively communicate the programme benefits and ensure that existing partners continue to collaborate and share data.

Prior to the cessation of UK COVID-19 funding, it was suggested that the NVAP should consider its future operating model. The idea of developing a vision and ambition for the NVAP was discussed; “*there is a need to think about the vision, the ambition, what the version 2.0 [of the NVAP] looks like”* (Interview Participant). Several interview participants discussed focusing on increased collaboration and innovation, but also acknowledged the importance of maintaining effective elements of the existing model (e.g., the bottom-up approach).

Several partners discussed further developing regional hubs in line with WHO pandemic preparedness strategies. There was consensus that regional hubs should be strengthened through technical assistance and training and the dissemination of the skills and expertise to countries within regions should be promoted. Broader strategic alignment was also discussed, with various UKHSA and Department of Health and Social Care (DHSC) global programmes suggested. Partners reflected that strategic partnerships and linking into broader programmes would reduce duplication, increase efficiency, improve visibility, and enhance the NVAP’s impact.

Funding concerns were a recurring theme regarding the NVAP’s sustainability. The year-on-year funding model and lack of guaranteed future funding meant that the programme was unable to make commitments to partners and plan future activities (NVAP documentation). Several partners reflected that sustainable funding was therefore needed and several funding options were outlined. The NVAP’s funding and future operating model should be aligned to “*increase the impact of the investment made in the building phase*” (Interview Participant).

The value of the NVAP’s capability and capacity strengthening offers were also highlighted. Several partners noted that the NVAP training and technical assistance offer should be continued and remained highly valuable in a rapidly evolving sequencing landscape. Future training session ideas were proposed, including metagenomics training and advanced training on bioinformatics. It was also suggested that there should be regular training sessions to ensure that knowledge and skills remain relevant, additional access to experts to address specific challenges, and in-person training sessions.

The NVAP’s SARS-CoV-2 support was valued by partners, but they advised that the NVAP should focus on enhancing its pathogen agnostic approach going forward:

“*As [the] NVAP has a remit for agnostic surveillance, I hope that the project becomes embedded to look at various other infectious diseases*” (Informant)

The NVAP had already begun to adopt a pathogen agnostic approach to supporting other countries with genomic sequencing of other pathogens (e.g., mpox). The NVAP also worked with bilateral partners to develop pathogen priority surveillance strategies and updated workplans (NVAP documentation).

The NVAP has contributed to enhancing global genomic surveillance through data sharing, development of global genomic surveillance tools, and collaboration. The data sharing established through the NVAP was a key achievement of the programme, and it was suggested that the NVAP should continue to focus on strengthening global data quality and timely reporting. One interview participant reflected that it was important to “*maximise data sharing benefits*”, for example by considering how the data can be used to support other priorities. Further improvements to data sharing practices will contribute to global genomic surveillance and pandemic preparedness through informing accurate risk assessments.

The collaborations developed between the NVAP and its partners was a strength of the programme and several partners voiced that they hoped the NVAP collaborations would evolve into longer term relationships. An interview participant noted that research and development opportunities with partners is an area of untapped potential, for example collaborations to develop vaccines and countermeasures for various pathogens. Partners also suggested that the NVAP could work with a greater range of partners, including philanthropic organisations and regional groups.

The NVAP has demonstrated the UK’s leadership in genomic surveillance. Establishing effective collaborations has enhanced this further. An interview participant noted that the NVAP should continue to engage globally to be recognised as a global genomic leader. An interview participant noted that engagement through global forums was also considered essential, particularly by contributing to and complementing the approaches of global organisations such as the IPSN.

Enacting the NVAP’s pandemic preparedness vision alongside developing new genomic surveillance tools and enhancing capacity was considered essential for improving global health security and pandemic preparedness. An informant acknowledged that the NVAP “*must keep doing good work for future pandemic preparedness and strengthen global surveillance and global health*”. This could be achieved through ensuring that the NVAP is adaptable to a range of pathogens, and putting processes, guidance, and agreements in place, as participants suggested. The NVAP should also consider the wider public health utility of genomic surveillance, for example developing countermeasures (including therapeutics, diagnostics, and vaccines) to “*make decisions about ongoing public health threats like AMR and tuberculosis*” (Interview Participant).

## Discussion

Genomic surveillance contributed significantly to global scientific understanding of COVID-19 which in turn informed the public health response [13]. Global genomic surveillance capability increased substantially during the pandemic; in February 2021, 103 of 194 WHO Member States (53%) had sequencing capacity for SARS-CoV-2, which increased to 163 of 194 WHO Member States (84%) by December 2022 [14]. Many countries leveraged the urgency of the pandemic to rapidly build their genomic surveillance capacity [15], supported by coordinated international action encompassing global, regional, and external partner (e.g., the NVAP) networks and initiatives [14]. However, the stability of the global genomic surveillance capability and capacity developed during the COVID-19 pandemic remains to be seen [14]. Further work will be required to sustain and expand genomic surveillance globally [14], particularly in low-and middle-income countries [1].

The NVAP was setup as part of the UK’s COVID-19 pandemic response to strengthen global genomic surveillance in countries with limited or no in-country capacity, share the UK’s genomics expertise globally, and strengthen national and global health security. The UK demonstrated high SARS-CoV-2 case sequencing rates and effective implementations of genomic surveillance programmes [16] and was therefore well placed to assist other countries through the NVAP.

There was evidence that the NVAP had delivered against objectives NVAP O1 (capability strengthening), NVAP O2 (capacity strengthening) and NVAP O5 (global genomic surveillance, global leadership and advocacy, and global health security and pandemic preparedness). More limited evidence was found against NVAP O3 (surge capacity) and NVAP O4 (biological risk assessment) due to limited requests for these from partners.

There was also evidence to support the NVAP theory of change model, which guided the programme’s development and implementation. However, despite the robust framework some of the proposed outcomes had not been fully achieved. For example, embedding genomics into frontline outbreak management and risk assessment and developing sustainable sequencing was an ongoing challenge. Additionally, due to the NVAP offering tailored support to partners, experiences of the programme outcomes and milestones varied between partners.

The primary impact of the NVAP was enabling partners to start undertaking genomic surveillance, or strengthening their existing capacity and capability, resulting in the provision of a comprehensive national or regional COVID-19 genomic sequencing service. It was evident that partners preferred to develop in-country and regional capacity and capability, rather than send samples to the UK for sequencing. The NVAP also demonstrated the UK’s global genomic leadership and improved preparedness for future pandemic and epidemic threats.

Partners reflected that the NVAP responded to their requirements throughout the SARS-CoV-2 pandemic, particularly through training and technical support. The NVAP was closely aligned with WHO and IPSN strategic priorities and workstreams, and partners supported the programme’s bottom-up, collaborative approach. A similar approach was employed by the Centre for Pathogen Genomics at the University of Melbourne, which supported genomic surveillance capacity building initiatives across a range of pathogens in eight countries in the Asia-Pacific Region [11]. Working closely with the WHO, promoting international collaboration, and information sharing were considered to be key components of successful international genomic surveillance programmes [11]. The UK’s COVID-19 Genomics UK (COG-UK) consortium made a key contribution to enhancing genomic sequencing in the UK during the pandemic [17]. An evaluation of this programme, which had a primarily domestic focus, outlined lessons also reflected in this evaluation, for example developing longer-term strategic plans, and maintaining genomic surveillance momentum [17].

The NVAP programme was closed on 31 March 2024 due to the public health management of COVID-19 being more aligned with other common respiratory illnesses and cessation of UK COVID-19 funding. However, learning from this evaluation should be applied to other global genomic surveillance programmes. Genomic surveillance strategies, including the WHO global genomic surveillance strategy for pathogens with pandemic and epidemic potential (2022–2032) [11] and UKHSA pathogen genomics strategy [18] outline the ongoing importance of investing in and maximizing the benefits of genomic surveillance to mitigate public health threats. This has also been reflected in the literature, with sustained investment in genomic surveillance strategies considered essential to identify the emergence of any potential viral pathogen or associated variants [19].

The importance of international collaboration and strengthening partnerships, with the benefits of reducing duplication and sharing expertise, was reflected throughout this evaluation. The NVAP programme was also included as an example of an effective partner initiative in the progress report on the first year of implementation of the WHO global genomic surveillance strategy [14]. This highlighted the NVAPs role in advancing global health objectives through strategic partnerships and collaborations. Continuing to develop global pathogen genomic surveillance capabilities through collaborations is a priority to improve pandemic preparedness [1]. Additionally, this evaluation highlighted the importance of exploring the untapped benefits of partnerships in future programmes, for example research and development opportunities, and responding to partner requirements through a comprehensive bottom-up approach.

There was consensus from evaluation participants and the wider literature that efforts to strengthen SARS-CoV-2 sequencing capability and capacity internationally, through programmes such as the NVAP, were essential during the pandemic response [14]. However, a pathogen agnostic strategy is essential for the ongoing utility and sustainability of genomic surveillance programmes [20,21]. This is reflected in the UKHSA pathogen genomics strategy, which highlights antimicrobial resistance (AMR), emerging infections and biosecurity, and vaccine-preventable diseases as future priority areas for genomic surveillance [18]. The WHO strategy also outlines the importance of genomic surveillance for other pathogens with pandemic or epidemic potential [11]. Planning, establishing, and supporting genomic pathogen surveillance structures will be crucial to identify and address future pandemics and infectious disease challenges [16]. Additionally, genomic surveillance capacity building initiatives should continue to be reinforced by locally tailored support [22].

The global data sharing established through the NVAP was highlighted as a key programme achievement, and sharing genomic data internationally remains critical [23]. This was also highlighted as a significant aspect of the WHO and UKHSA genomic surveillance strategies [11,18]. Data sharing must be underpinned by institutional governance and technical-standard setting to promote timely, ethical, accurate and coherent data sharing across the global genomic surveillance network [22]. The impact of genomic data is dependent on high quality and timely data [24] contextualised with clinical data [25]. Continuing to promote global data sharing and GISAID uploads, as well as enhancing the timeliness and quality of data is important for the impact and sustainability of future global genomic surveillance programmes.

This rapid evaluation involved a mixed-methods approach which provided a comprehensive overview of how the NVAP was developed, delivered, and experienced by partners. A broad range of stakeholders were involved, and the majority were highly engaged with the evaluation data collection process. However, we identified several limitations. Survey or interview responses were not received from all the NVAP partners despite sending several reminders and extending the survey and interview deadlines, and therefore their views and experiences were not captured in this evaluation. Feedback was not obtained from countries or regions that did not proceed with the NVAP support or had received targeted support (e.g., surge capacity support), and we cannot exclude the potential that programme-level outcomes may be subject to some bias. Furthermore, some stakeholders only experienced or contributed to a single aspect of the NVAP (e.g., delivering training) so their responses were limited. The evaluation was conducted using rapid methods, which restricted the scope of this evaluation to explore additional areas, and the structured interviews offered limited flexibility to explore additional emerging themes. It was challenging to define performance against the overarching NVAP objectives due to their broad nature, and objectives and deliverables for the individual NVAP partners were not reviewed as part of this evaluation. Finally, it was challenging to measure public health outcomes directly linked to the NVAP or the cost-effectiveness of the programme.

## Conclusion

The NVAP was a successful intervention which achieved its overarching objectives and offered tangible benefits for bilateral and regional partners, the UK’s reputation, and global health security. The NVAP was UKHSA’s flagship global genomic surveillance programme, and there was consensus that the offer was unique and highly responsive to partner needs. However, the programme was closed on 31 March 2024 due to the public health management of COVID-19 being more aligned with other common respiratory illnesses. This evaluation presented the NVAP’s successes and limitations, as well as important and timely lessons that can be applied to future global genomic surveillance programmes. Moving forward, global health stakeholders should build resilient and adaptive surveillance systems, ensuring rapid detection and response to future health threats. Future research should focus on the longer-term utility and sustainability of global genomic surveillance initiatives and programmes. By continuing to build and strengthen alliances, the global community can better prepare for and respond to future public health challenges.

## Supporting information

S1 Survey NVAP Bilateral Partner Survey. (HTML)

S2 Survey NVAP Regional Partner Survey. (HTML)

S3 Survey NVAP Informant Survey. (HTML)
