# Supplementary material for: A rapid evaluation of the UK Health Security Agency’s New Variant Assessment Platform global genomic surveillance programme
Source: PLOS Glob Public Health. 2025 Dec 5;5(12):e0005578. doi: 10.1371/journal.pgph.0005578 (PMC12680177; doi:10.1371/journal.pgph.0005578)
Supplement: S1 Survey — (HTML) [file pgph.0005578.s001.html]

 NVAP Country Partner Survey 


|  |
| --- |
|  |
|  |
| NVAP Country Partner Survey |

|  |  |  |  |  |  |  |  |  |  |  |  |  |  |  |  |  |  |  |  |  |  |  |  |  |  |  |  |  |  |  |  |  |  |  |  |  |  |  |  |  |  |  |
| --- | --- | --- | --- | --- | --- | --- | --- | --- | --- | --- | --- | --- | --- | --- | --- | --- | --- | --- | --- | --- | --- | --- | --- | --- | --- | --- | --- | --- | --- | --- | --- | --- | --- | --- | --- | --- | --- | --- | --- | --- | --- | --- |
| |  | | --- | |  | | |  |  |  | | --- | --- | --- | |  | | | |  | Background + Section 1: Country/Institution Details |  | | NVAP Background The New Variant Assessment Platform (NVAP) was launched by the UK Health Security Agency (UKHSA) in April 2021 as part of the COVID-19 pandemic response to support other countries to detect and report new variants of SARS-CoV-2 rapidly. The programme is in its third year of funding and is now focussed on a pathogen agnostic strategy to strengthen genomic surveillance globally. NVAP supports partners in several ways, including genomic sequencing, bioinformatic assessment, characterisation of variants, risk assessment and immunological testing of detected variants of concern (VOCs).   NVAP Evaluation UKHSA’s NVAP team and Evaluation and Epidemiological Science (EES) Division are collaborating on a rapid evaluation of the NVAP programme. This will be the first structured evaluation of the NVAP programme to date and will be completed by the end of October 2023. It is timely to evaluate the NVAP programme to enable contribution to decisions regarding ongoing activity, potential expansion, and future funding beyond March 2024.    NVAP Evaluation Survey This survey is being conducted with key partners who are working with the NVAP team to deliver the collaboration agreement objectives. We would appreciate your input into the evaluation, and survey feedback will be used to understand the impact that NVAP has had in strengthening genomic sequencing in your country/institution, experiences of co-developing and delivering NVAP, the lessons learnt, examples of programme strengths and limitations, and the future expectations of the programme.   Please can you complete this survey by Friday 1st September 2023. If you think there is someone else in your country/institution who can also provide feedback to this survey, please forward it to them. This survey should take no longer than 45 minutes to complete. The survey can be closed during completion, and your responses will be saved. You will be able to return to the first uncompleted page of the survey when you click on the survey link, but you will not be able to amend your answers once the survey has been submitted.  The answers given will be kept in strict confidence and will be held and processed securely in line with the Data Protection Act 2018 and UKHSA information governance policies and procedures. Reporting of the findings will be anonymised so that individual responses cannot be identified.  If you have any queries about this survey or the evaluation, please contact UKHSA’s Evaluation and Epidemiological Science team via email: evaluationepiscience@ukhsa.gov.uk, quoting the reference ‘NVAP Evaluation’ in the email header.   Section 1 This section captures information about your country/institution. |  |  |  |  |  |  |  |  |  |  |  |  |  |  |  |  |  |  |  |  |  |  |  |  |  |  |  |  |  |  |  |  |  |  | | --- | --- | --- | --- | --- | --- | --- | --- | --- | --- | --- | --- | --- | --- | --- | --- | --- | --- | --- | --- | --- | --- | --- | --- | --- | --- | --- | --- | --- | --- | --- | --- | --- | |  | |  |  | | --- | --- | | 1. | Country\* | |  |  | |  |  |  |  |  | | --- | --- | | 2. | Institution (please include multiple if appropriate) | |  | |  |  | | --- | --- | | 1 |  | | 2 |  | | 3 |  | | |  |  |  |  |  | | --- | --- | | 3. | Full name of respondent\* | |  |  | |  |  |  |  |  | | --- | --- | | 4. | Job title of respondent\* | |  |  | |  |  | |  | | |

|  |
| --- |
|  |
|  |
| NVAP Country Partner Survey |

|  |  |  |  |  |  |  |  |  |  |  |  |  |  |  |  |  |  |  |  |  |  |  |  |  |  |  |  |  |  |  |  |  |  |  |  |  |  |  |  |  |  |  |  |  |  |  |  |  |  |  |  |  |  |  |  |  |  |  |  |  |  |  |  |  |  |  |  |  |  |  |  |  |  |  |  |  |  |  |  |  |  |  |  |  |  |  |  |  |  |  |  |  |  |  |  |  |  |  |  |  |  |  |  |
| --- | --- | --- | --- | --- | --- | --- | --- | --- | --- | --- | --- | --- | --- | --- | --- | --- | --- | --- | --- | --- | --- | --- | --- | --- | --- | --- | --- | --- | --- | --- | --- | --- | --- | --- | --- | --- | --- | --- | --- | --- | --- | --- | --- | --- | --- | --- | --- | --- | --- | --- | --- | --- | --- | --- | --- | --- | --- | --- | --- | --- | --- | --- | --- | --- | --- | --- | --- | --- | --- | --- | --- | --- | --- | --- | --- | --- | --- | --- | --- | --- | --- | --- | --- | --- | --- | --- | --- | --- | --- | --- | --- | --- | --- | --- | --- | --- | --- | --- | --- | --- | --- | --- | --- |
| |  | | --- | |  | | |  |  |  | | --- | --- | --- | |  | | | |  | Section 2: Capability strengthening through technical assistance and training for pathogen genomic surveillance |  | | This section covers your experience with the NVAP programme offers related to capability strengthening in the form of upskilling staff and providing technical guidance for streamlining the end-to-end genomic sequencing process. |  |  |  |  |  |  |  |  |  |  |  |  |  |  |  |  |  |  |  |  |  |  |  |  |  |  |  |  |  |  |  |  |  |  |  |  |  |  |  |  |  |  |  |  |  |  |  |  |  |  |  |  |  |  |  |  |  |  |  |  |  |  |  |  |  |  |  |  |  |  |  |  |  |  |  |  |  |  |  |  |  |  |  |  |  |  |  |  |  |  |  |  |  |  |  | | --- | --- | --- | --- | --- | --- | --- | --- | --- | --- | --- | --- | --- | --- | --- | --- | --- | --- | --- | --- | --- | --- | --- | --- | --- | --- | --- | --- | --- | --- | --- | --- | --- | --- | --- | --- | --- | --- | --- | --- | --- | --- | --- | --- | --- | --- | --- | --- | --- | --- | --- | --- | --- | --- | --- | --- | --- | --- | --- | --- | --- | --- | --- | --- | --- | --- | --- | --- | --- | --- | --- | --- | --- | --- | --- | --- | --- | --- | --- | --- | --- | --- | --- | --- | --- | --- | --- | --- | --- | --- | --- | --- | --- | --- | |  | |  |  | | --- | --- | | 5. | Has your country/institution received capability strengthening support for pathogen genomic sequencing as part of the NVAP programme?\* | |  | Yes No | |  |  |  |  |  | | --- | --- | | 6. | What support has your country/institution received as part of your collaboration with NVAP? Please select all that apply\* | |  | |  | | --- | | Technical assistance from the NVAP team | | NVAP training sessions | | Knowledge exchange sessions with technical experts from across UKHSA/other partners | | Technical assistance for External Quality Assessment for SARS-CoV-2 |  Other (please specify) | |  |  |  |  |  | | --- | --- | |  |  |  |  |  | | --- | --- | | 7. | Please specify what NVAP technical assistance your country/institution has accessed? Please select all that apply\* | |  | |  | | --- | | Bioinformatic analytical support | | Sequencing protocols | | Epidemiological support | | Genomic surveillance strategy development | | Guidance development | | Troubleshooting |  Other (please specify) | |  |  |  |  |  | | --- | --- | |  |  |  |  |  | | --- | --- | | 8. | Please can you specify which NVAP training sessions your country/institution have attended? Please select all that apply\* | |  | |  | | --- | | Variant risk assessment training | | Virtual bioinformatics training | | In country sequencing EQA | | Variant epidemiology training | | Metagenomics | | |  |  |  |  |  | | --- | --- | |  |  |  |  |  | | --- | --- | | 9. | How satisfied are you with the NVAP capability strengthening support for pathogen genomic sequencing?\* | |  | |  | | --- | | Very satisfied | | Satisfied | | Neutral | | Slightly satisfied | | Not satisfied |  |  | | --- | |  | | |  |  |  |  |  | | --- | --- | |  |  |  |  |  | | --- | --- | | 10. | How would you rate your access to NVAP capacity and capability strengthening support (e.g., timely and regular communication, IT connectivity, time difference, resolving technical queries etc.)?\* | |  | |  | | --- | | Very accessible | | Accessible | | Neutral | | Slightly accessible | | Not accessible |  |  | | --- | |  | | |  |  |  |  |  | | --- | --- | |  |  |  |  |  | | --- | --- | | 11. | Please describe the benefits of the NVAP capability strengthening support offer for your country/institution.\* | |  |  | |  |  |  |  |  | | --- | --- | | 12. | Please describe the limitations of the NVAP capability strengthening support offer for your country/institution.\* | |  |  | |  |  |  |  |  | | --- | --- | | 13. | Please outline the additional capability strengthening support that you would like the NVAP programme to provide for your country/institution in the future. | |  |  | |  |  | |  | | |

|  |
| --- |
|  |
|  |
| NVAP Country Partner Survey |

|  |  |  |  |  |  |  |  |  |  |  |  |  |  |  |  |  |  |  |  |  |  |  |  |  |  |  |  |  |  |  |  |  |  |  |  |  |  |  |  |  |  |  |  |  |  |  |  |  |  |  |  |  |  |  |  |  |  |  |  |  |  |  |  |  |  |  |  |  |  |  |  |  |  |  |  |  |  |  |  |  |  |  |  |  |  |  |  |  |  |  |  |
| --- | --- | --- | --- | --- | --- | --- | --- | --- | --- | --- | --- | --- | --- | --- | --- | --- | --- | --- | --- | --- | --- | --- | --- | --- | --- | --- | --- | --- | --- | --- | --- | --- | --- | --- | --- | --- | --- | --- | --- | --- | --- | --- | --- | --- | --- | --- | --- | --- | --- | --- | --- | --- | --- | --- | --- | --- | --- | --- | --- | --- | --- | --- | --- | --- | --- | --- | --- | --- | --- | --- | --- | --- | --- | --- | --- | --- | --- | --- | --- | --- | --- | --- | --- | --- | --- | --- | --- | --- | --- | --- | --- |
| |  | | --- | |  | | |  |  |  | | --- | --- | --- | |  | | | |  | Section 3: Capacity strengthening for pathogen surveillance |  | | This section covers your experience with the NVAP programme offers related to capacity strengthening in the form of procurement of sequencing infrastructure and assistance/guidance on streamlining sequencing protocols, analysis and reporting to increase sequencing throughput. |  |  |  |  |  |  |  |  |  |  |  |  |  |  |  |  |  |  |  |  |  |  |  |  |  |  |  |  |  |  |  |  |  |  |  |  |  |  |  |  |  |  |  |  |  |  |  |  |  |  |  |  |  |  |  |  |  |  |  |  |  |  |  |  |  |  |  |  |  |  |  |  |  |  |  |  |  |  |  |  |  |  |  | | --- | --- | --- | --- | --- | --- | --- | --- | --- | --- | --- | --- | --- | --- | --- | --- | --- | --- | --- | --- | --- | --- | --- | --- | --- | --- | --- | --- | --- | --- | --- | --- | --- | --- | --- | --- | --- | --- | --- | --- | --- | --- | --- | --- | --- | --- | --- | --- | --- | --- | --- | --- | --- | --- | --- | --- | --- | --- | --- | --- | --- | --- | --- | --- | --- | --- | --- | --- | --- | --- | --- | --- | --- | --- | --- | --- | --- | --- | --- | --- | --- | --- | |  | |  |  | | --- | --- | | 14. | Has your country/institution received NVAP support for increasing sequencing throughput to strengthen your sequencing capacity?\* | |  | Yes No | |  |  |  |  |  | | --- | --- | | 15. | What support has your country/institution received to date as part of the NVAP programme? Please select all that apply.\* | |  | |  | | --- | | Procurement of reagents | | Procurement of laboratory equipment and consumables | | Procurement of computing infrastructure (e.g. servers, desktops) | | Registration and procurement for External Quality Assessment scheme for SARS-CoV-2 | | Guidance and training on sequencing workflows and bioinformatic pipelines |  Other (please specify) | |  |  |  |  |  | | --- | --- | |  |  |  |  |  | | --- | --- | | 16. | How satisfied are you with the NVAP support for increasing your sequencing throughput to strengthen your sequencing capacity?\* | |  | |  | | --- | | Very satisfied | | Satisfied | | Neutral | | Slightly satisfied | | Not satisfied |  |  | | --- | |  | | |  |  |  |  |  | | --- | --- | |  |  |  |  |  | | --- | --- | | 17. | Please estimate your next generation sequencing (NGS) capacity for pathogen genomics per month prior to receiving NVAP support.\* | |  |  | |  |  |  |  |  | | --- | --- | | 18. | Please estimate your current NGS sequencing capacity for pathogen genomics per month after receiving NVAP support.\* | |  |  | |  |  |  |  |  | | --- | --- | | 19. | If there has been a change in NGS sequencing capacity for pathogen genomics following NVAP support, please select all the reasons that apply.\* | |  | |  | | --- | | Procurement of reagents | | Procurement of equipment/consumables | | Technical assistance or guidance | | Procurement of IT infrastructure | | Training and follow up support in bioinformatic analysis | | Not applicable - there has been no change |  Other (please specify) | |  |  |  |  |  | | --- | --- | |  |  | |  |  |  |  |  | | --- | --- | | 20. | Please describe the benefits of the support provided by the NVAP programme to strengthen sequencing capacity for your country/institution\* | |  |  | |  |  |  |  |  | | --- | --- | | 21. | Please describe the limitations of the support provided by the NVAP programme to strengthen sequencing capacity for your country/institution.\* | |  |  | |  |  |  |  |  | | --- | --- | | 22. | Please outline any additional support to increase sequencing throughput that you would like the NVAP programme to provide for your country/institution in the future. | |  |  | |  |  | |  | | |

|  |
| --- |
|  |
|  |
| NVAP Country Partner Survey |

|  |  |  |  |  |  |  |  |  |  |  |  |  |  |  |  |  |  |  |  |  |  |  |  |  |  |  |  |  |  |  |  |  |  |  |  |  |  |  |  |  |  |  |  |  |
| --- | --- | --- | --- | --- | --- | --- | --- | --- | --- | --- | --- | --- | --- | --- | --- | --- | --- | --- | --- | --- | --- | --- | --- | --- | --- | --- | --- | --- | --- | --- | --- | --- | --- | --- | --- | --- | --- | --- | --- | --- | --- | --- | --- | --- |
| |  | | --- | |  | | |  |  |  | | --- | --- | --- | |  | | | |  | Section 4: UK Sequencing Support |  | | This section covers your experience with the NVAP UK sequencing support offer that includes sequencing of samples in the UK and subsequent support for bioinformatic analysis and interpretation. |  |  |  |  |  |  |  |  |  |  |  |  |  |  |  |  |  |  |  |  |  |  |  |  |  |  |  |  |  |  |  |  |  |  |  |  | | --- | --- | --- | --- | --- | --- | --- | --- | --- | --- | --- | --- | --- | --- | --- | --- | --- | --- | --- | --- | --- | --- | --- | --- | --- | --- | --- | --- | --- | --- | --- | --- | --- | --- | --- | |  | |  |  | | --- | --- | | 23. | Has your country/institution requested UK sequencing support as part of the NVAP offer?\* | |  | Yes No | |  |  |  |  |  | | --- | --- | | 24. | How many samples has your country/institution sent to the UK via NVAP for sequencing support?\* | |  |  | |  |  |  |  |  | | --- | --- | | 25. | How satisfied are you with the sequencing support provided by the NVAP programme?\* | |  | |  | | --- | | Very satisfied | | Satisfied | | Neutral | | Slightly satisfied | | Not satisfied |  |  | | --- | |  | | |  |  |  |  |  | | --- | --- | |  |  |  |  |  | | --- | --- | | 26. | Please describe the benefits and limitations of the sequencing support provided by the NVAP programme for your country/institution\* | |  |  | |  |  | |  | | |

|  |
| --- |
|  |
|  |
| NVAP Country Partner Survey |

|  |  |  |  |  |  |  |  |  |  |  |  |  |  |  |  |  |  |  |  |  |  |  |  |  |  |  |  |  |  |  |  |  |  |  |  |  |  |  |  |  |  |  |  |  |  |  |  |  |  |  |
| --- | --- | --- | --- | --- | --- | --- | --- | --- | --- | --- | --- | --- | --- | --- | --- | --- | --- | --- | --- | --- | --- | --- | --- | --- | --- | --- | --- | --- | --- | --- | --- | --- | --- | --- | --- | --- | --- | --- | --- | --- | --- | --- | --- | --- | --- | --- | --- | --- | --- | --- |
| |  | | --- | |  | | |  |  |  | | --- | --- | --- | |  | | | |  | Section 5: Biological Risk Assessment for New Variants of SARS-CoV-2 |  | | This section covers your experience with sending samples to the UK via the NVAP programme for biological risk assessment for further variant characterization analysis or scientific exchange on neutralization study. |  |  |  |  |  |  |  |  |  |  |  |  |  |  |  |  |  |  |  |  |  |  |  |  |  |  |  |  |  |  |  |  |  |  |  |  |  |  |  |  |  |  | | --- | --- | --- | --- | --- | --- | --- | --- | --- | --- | --- | --- | --- | --- | --- | --- | --- | --- | --- | --- | --- | --- | --- | --- | --- | --- | --- | --- | --- | --- | --- | --- | --- | --- | --- | --- | --- | --- | --- | --- | --- | |  | |  |  | | --- | --- | | 27. | Has your country/institution received support with biological risk assessment for pathogens of concern as part of the NVAP programme? \* | |  | Yes No | |  |  |  |  |  | | --- | --- | | 28. | Please describe the support with biological risk assessment for pathogens of concern that your country/institution has received as part of the NVAP programme\* | |  |  | |  |  |  |  |  | | --- | --- | | 29. | How satisfied are you with the support for biological risk assessment for pathogens of concern provided by the NVAP programme?\* | |  | |  | | --- | | Very satisfied | | Satisfied | | Neutral | | Slightly satisfied | | Not satisfied |  |  | | --- | |  | | |  |  |  |  |  | | --- | --- | |  |  |  |  |  | | --- | --- | | 30. | Please describe the benefits and limitations of the biological risk assessment for pathogens of concern support provided by the NVAP programme for your country/institution\* | |  |  | |  |  |  |  |  | | --- | --- | | 31. | Please outline any additional support for biological risk assessment for pathogens of concern that you would like the NVAP programme to provide for your country/institution in the future | |  |  | |  |  | |  | | |

|  |
| --- |
|  |
|  |
| NVAP Country Partner Survey |

|  |  |  |  |  |  |  |  |  |  |  |  |  |  |  |  |  |  |  |  |  |  |  |  |  |  |  |  |  |  |  |  |  |  |  |  |  |  |  |  |  |  |  |  |  |  |  |  |  |  |  |  |  |  |  |  |  |  |  |  |  |  |  |  |  |  |  |  |  |  |  |  |  |  |  |  |
| --- | --- | --- | --- | --- | --- | --- | --- | --- | --- | --- | --- | --- | --- | --- | --- | --- | --- | --- | --- | --- | --- | --- | --- | --- | --- | --- | --- | --- | --- | --- | --- | --- | --- | --- | --- | --- | --- | --- | --- | --- | --- | --- | --- | --- | --- | --- | --- | --- | --- | --- | --- | --- | --- | --- | --- | --- | --- | --- | --- | --- | --- | --- | --- | --- | --- | --- | --- | --- | --- | --- | --- | --- | --- | --- | --- |
| |  | | --- | |  | | |  |  |  | | --- | --- | --- | |  | | | |  | Section 6: NVAP Views and Experiences |  | | This section covers your overall experience with the NVAP programme to date |  |  |  |  |  |  |  |  |  |  |  |  |  |  |  |  |  |  |  |  |  |  |  |  |  |  |  |  |  |  |  |  |  |  |  |  |  |  |  |  |  |  |  |  |  |  |  |  |  |  |  |  |  |  |  |  |  |  |  |  |  |  |  |  |  |  |  | | --- | --- | --- | --- | --- | --- | --- | --- | --- | --- | --- | --- | --- | --- | --- | --- | --- | --- | --- | --- | --- | --- | --- | --- | --- | --- | --- | --- | --- | --- | --- | --- | --- | --- | --- | --- | --- | --- | --- | --- | --- | --- | --- | --- | --- | --- | --- | --- | --- | --- | --- | --- | --- | --- | --- | --- | --- | --- | --- | --- | --- | --- | --- | --- | --- | --- | |  | |  |  | | --- | --- | | 32. | Was your country/institution involved in developing the original and ongoing collaboration agreement and work plan objectives with NVAP for partnership?\* | |  | Yes No | |  |  |  |  |  | | --- | --- | | 33. | Please rate the implementation and ongoing delivery of the NVAP programme to date in your country/institution\* | |  | |  | | --- | | Significantly exceeded expectations | | Exceeded expectations | | Met expectations | | Required some improvements | | Required significant improvements |  |  | | --- | |  | | |  |  |  |  |  | | --- | --- | |  |  |  |  |  | | --- | --- | | 34. | What have been the main impacts of the NVAP programme for your country/institution? Please select all that apply\* | |  | |  | | --- | | Improved sequencing services | | Streamlining of sequencing workflows | | Improved access to sequencing training and/or expertise | | Upskilling of bioinformatics staff | | Improved use of NGS data for surveillance and/or outbreak response |  Other (please specify) | |  |  |  |  |  | | --- | --- | |  |  |  |  |  | | --- | --- | | 35. | What have been the main benefits of the NVAP programme for your country/institution?\* | |  |  | |  |  |  |  |  | | --- | --- | | 36. | What have been the main limitations of the NVAP programme for your country/institution?\* | |  |  | |  |  |  |  |  | | --- | --- | | 37. | What additional support would you like the NVAP programme to provide for your country/institution in the future? | |  |  | |  |  |  |  |  | | --- | --- | | 38. | Please highlight **two** examples of NVAP support delivered in your country/organisation | |  |  | |  |  |  |  |  | | --- | --- | | 39. | Do you have any other comments or feedback on the NVAP programme? | |  |  | |  |  | |  | | |
