# Supplementary material for: A rapid evaluation of the UK Health Security Agency’s New Variant Assessment Platform global genomic surveillance programme
Source: PLOS Glob Public Health. 2025 Dec 5;5(12):e0005578. doi: 10.1371/journal.pgph.0005578 (PMC12680177; doi:10.1371/journal.pgph.0005578)
Supplement: S2 Survey — (HTML) [file pgph.0005578.s002.html]

 NVAP Regional Partner Survey 


|  |
| --- |
|  |
|  |
| NVAP Regional Partner Survey |

|  |  |  |  |  |  |  |  |  |  |  |  |  |  |  |  |  |  |  |  |  |  |  |  |  |  |  |  |  |  |  |  |  |  |  |  |  |  |  |  |  |  |  |  |  |
| --- | --- | --- | --- | --- | --- | --- | --- | --- | --- | --- | --- | --- | --- | --- | --- | --- | --- | --- | --- | --- | --- | --- | --- | --- | --- | --- | --- | --- | --- | --- | --- | --- | --- | --- | --- | --- | --- | --- | --- | --- | --- | --- | --- | --- |
| |  | | --- | |  | | |  |  |  | | --- | --- | --- | |  | | | |  | Background + Section 1: Region/Institution Details |  | | NVAP Background The New Variant Assessment Platform (NVAP) was launched by the UK Health Security Agency (UKHSA) in April 2021 as part of the COVID-19 pandemic response to support other countries to detect and report new variants of SARS-CoV-2 rapidly. The programme is in its third year of funding and is now focussed on a pathogen agnostic strategy to strengthen genomic surveillance globally. NVAP supports partners in several ways, including genomic sequencing, bioinformatic assessment, characterisation of variants, risk assessment and immunological testing of detected variants of concern (VOCs).   NVAP Evaluation UKHSA’s NVAP team and Evaluation and Epidemiological Science (EES) Division are collaborating on a rapid evaluation of the NVAP programme. This will be the first structured evaluation of the NVAP programme to date and will be completed by the end of October 2023. It is timely to evaluate the NVAP programme to enable contribution to decisions regarding ongoing activity, potential expansion, and future funding beyond March 2024.    NVAP Evaluation Survey This survey is being conducted with key partners who are working with the NVAP team to deliver the collaboration agreement objectives. We would appreciate your input into the evaluation, and survey feedback will be used to understand the impact that NVAP has had in strengthening genomic sequencing in your region/institution, experiences of co-developing and delivering NVAP, the lessons learnt, examples of programme strengths and limitations, and the future expectations of the programme.   Please can you complete this survey by Friday 1st September 2023. If you think there is someone else in your region/institution who can also provide feedback to this survey, please forward it to them. This survey should take no longer than 20 minutes to complete. The survey can be closed during completion, and your responses will be saved. You will be able to return to the first uncompleted page of the survey when you click on the survey link, but you will not be able to amend your answers once the survey has been submitted.   The answers given will be kept in strict confidence and will be held and processed securely in line with the Data Protection Act 2018 and UKHSA information governance policies and procedures. Reporting of the findings will be anonymised so that individual responses cannot be identified.  If you have any queries about this survey or the evaluation, please contact UKHSA’s Evaluation and Epidemiological Science team via email: evaluationepiscience@ukhsa.gov.uk, quoting the reference ‘NVAP Evaluation’ in the email header.   Section 1: Region/Institution Details This section captures information about your region/institution |  |  |  |  |  |  |  |  |  |  |  |  |  |  |  |  |  |  |  |  |  |  |  |  |  |  |  |  |  |  |  |  |  |  |  |  | | --- | --- | --- | --- | --- | --- | --- | --- | --- | --- | --- | --- | --- | --- | --- | --- | --- | --- | --- | --- | --- | --- | --- | --- | --- | --- | --- | --- | --- | --- | --- | --- | --- | --- | --- | |  | |  |  | | --- | --- | | 1. | Region\* | |  |  | |  |  |  |  |  | | --- | --- | | 2. | Institution (include multiple if appropriate) | |  | |  |  | | --- | --- | | 1 |  | | At least 1 row is required in this question type. |  | | 2 |  | | 3 |  | | |  |  |  |  |  | | --- | --- | | 3. | Full name of respondent\* | |  |  | |  |  |  |  |  | | --- | --- | | 4. | Job title of respondent\* | |  |  | |  |  | |  | | |

|  |
| --- |
|  |
|  |
| NVAP Regional Partner Survey |

|  |  |  |  |  |  |  |  |  |  |  |  |  |  |  |  |  |  |  |  |  |  |  |  |  |  |  |  |  |  |  |  |  |  |  |  |  |  |  |  |  |  |  |  |  |  |  |  |  |  |  |  |  |  |  |  |  |  |  |  |  |  |  |  |  |  |  |  |  |  |  |  |  |  |  |  |  |  |  |  |  |  |  |  |  |  |  |  |  |  |  |  |  |  |  |  |  |  |  |  |  |  |  |  |  |  |  |  |  |  |  |  |  |
| --- | --- | --- | --- | --- | --- | --- | --- | --- | --- | --- | --- | --- | --- | --- | --- | --- | --- | --- | --- | --- | --- | --- | --- | --- | --- | --- | --- | --- | --- | --- | --- | --- | --- | --- | --- | --- | --- | --- | --- | --- | --- | --- | --- | --- | --- | --- | --- | --- | --- | --- | --- | --- | --- | --- | --- | --- | --- | --- | --- | --- | --- | --- | --- | --- | --- | --- | --- | --- | --- | --- | --- | --- | --- | --- | --- | --- | --- | --- | --- | --- | --- | --- | --- | --- | --- | --- | --- | --- | --- | --- | --- | --- | --- | --- | --- | --- | --- | --- | --- | --- | --- | --- | --- | --- | --- | --- | --- | --- | --- | --- | --- | --- |
| |  | | --- | |  | | |  |  |  | | --- | --- | --- | |  | | | |  | Section 2: Regional capability and capacity strengthening through technical assistance and training for pathogen genomic surveillance |  | | This section covers your experience with the NVAP programme offers related to regional capacity and capability strengthening in the form of upskilling staff from national public institutions in your region, providing bespoke training and technical guidance for streamlining the end to end genomic sequencing process. |  |  |  |  |  |  |  |  |  |  |  |  |  |  |  |  |  |  |  |  |  |  |  |  |  |  |  |  |  |  |  |  |  |  |  |  |  |  |  |  |  |  |  |  |  |  |  |  |  |  |  |  |  |  |  |  |  |  |  |  |  |  |  |  |  |  |  |  |  |  |  |  |  |  |  |  |  |  |  |  |  |  |  |  |  |  |  |  |  |  |  |  |  |  |  |  |  |  |  |  |  |  |  |  | | --- | --- | --- | --- | --- | --- | --- | --- | --- | --- | --- | --- | --- | --- | --- | --- | --- | --- | --- | --- | --- | --- | --- | --- | --- | --- | --- | --- | --- | --- | --- | --- | --- | --- | --- | --- | --- | --- | --- | --- | --- | --- | --- | --- | --- | --- | --- | --- | --- | --- | --- | --- | --- | --- | --- | --- | --- | --- | --- | --- | --- | --- | --- | --- | --- | --- | --- | --- | --- | --- | --- | --- | --- | --- | --- | --- | --- | --- | --- | --- | --- | --- | --- | --- | --- | --- | --- | --- | --- | --- | --- | --- | --- | --- | --- | --- | --- | --- | --- | --- | --- | --- | --- | |  | |  |  | | --- | --- | | 5. | Has your region/institution received capacity and capability strengthening support for pathogen genomic sequencing as part of the NVAP programme?\* | |  | Yes No | |  |  |  |  |  | | --- | --- | | 6. | What support has your region/institution received as part of your collaboration with NVAP? Please select all that apply\* | |  | |  | | --- | | Strengthening of regional hubs | | Scoping of pathogen genomic surveillance capacity in the region | | Technical assistance from NVAP | | Access to NVAP training | | Knowledge exchange sessions with technical experts from across UKHSA/other partners | | Deployment of UKHSA technical specialist advisor | | Establishment of External Quality Assessment for SARS-CoV-2 | | Regional genomic surveillance strategy or roadmap development |  Other (please specify) | |  |  |  |  |  | | --- | --- | |  |  |  |  |  | | --- | --- | | 7. | Please can you specify which NVAP training sessions your region/institution have attended? Please select all that apply\* | |  | |  | | --- | | Variant risk assessment training | | Virtual bioinformatics training | | In country sequencing EQA and bioinformatics training | | Variant epidemiology training | | Metagenomics | | |  |  |  |  |  | | --- | --- | |  |  |  |  |  | | --- | --- | | 8. | Please can you specify what NVAP technical assistance your region/institution have accessed? Please select all that apply\* | |  | |  | | --- | | Bioinformatic analytical support | | Sequencing protocols | | Epidemiological support | | Guidance development | | Troubleshooting |  Other (please specify) | |  |  |  |  |  | | --- | --- | |  |  |  |  |  | | --- | --- | | 9. | Please describe how the collaboration with the deployed staff works in your region/institution (including benefits and limitations)?\* | |  |  | |  |  |  |  |  | | --- | --- | | 10. | Please describe your experience of the deployment process (please elaborate on what worked and what proved to be a challenge)\* | |  |  | |  |  |  |  |  | | --- | --- | | 11. | How satisfied are you with the NVAP capability and capacity strengthening support for pathogen genomic sequencing?\* | |  | |  | | --- | | Very satisfied | | Satisfied | | Neutral | | Slightly satisfied | | Not satisfied |  |  | | --- | |  | | |  |  |  |  |  | | --- | --- | |  |  |  |  |  | | --- | --- | | 12. | How would you rate your access to NVAP capacity and capability strengthening support (e.g., timely and regular communication, IT connectivity, time difference, resolving technical queries etc.)?\* | |  | |  | | --- | | Very accessible | | Accessible | | Neutral | | Slightly accessible | | Not accessible |  |  | | --- | |  | | |  |  |  |  |  | | --- | --- | |  |  |  |  |  | | --- | --- | | 13. | Please describe the benefits of the NVAP capacity and capability strengthening support offer for your region/institution \* | |  |  | |  |  |  |  |  | | --- | --- | | 14. | Please describe the limitations of the NVAP capacity and capability strengthening support offer for your region/institution\* | |  |  | |  |  | |  | | |

|  |
| --- |
|  |
|  |
| NVAP Regional Partner Survey |

|  |  |  |  |  |  |  |  |  |  |  |  |  |  |  |  |  |  |  |  |  |  |  |  |  |  |  |  |  |  |  |  |  |  |  |  |  |  |  |  |  |  |  |  |  |  |  |  |  |  |  |  |  |  |  |  |  |  |  |  |  |  |  |  |  |  |  |  |  |  |  |  |  |  |  |  |
| --- | --- | --- | --- | --- | --- | --- | --- | --- | --- | --- | --- | --- | --- | --- | --- | --- | --- | --- | --- | --- | --- | --- | --- | --- | --- | --- | --- | --- | --- | --- | --- | --- | --- | --- | --- | --- | --- | --- | --- | --- | --- | --- | --- | --- | --- | --- | --- | --- | --- | --- | --- | --- | --- | --- | --- | --- | --- | --- | --- | --- | --- | --- | --- | --- | --- | --- | --- | --- | --- | --- | --- | --- | --- | --- | --- |
| |  | | --- | |  | | |  |  |  | | --- | --- | --- | |  | | | |  | Section 3: NVAP Views and Experiences |  | | This section covers your overall experience with the NVAP programme to date |  |  |  |  |  |  |  |  |  |  |  |  |  |  |  |  |  |  |  |  |  |  |  |  |  |  |  |  |  |  |  |  |  |  |  |  |  |  |  |  |  |  |  |  |  |  |  |  |  |  |  |  |  |  |  |  |  |  |  |  |  |  |  |  |  |  |  | | --- | --- | --- | --- | --- | --- | --- | --- | --- | --- | --- | --- | --- | --- | --- | --- | --- | --- | --- | --- | --- | --- | --- | --- | --- | --- | --- | --- | --- | --- | --- | --- | --- | --- | --- | --- | --- | --- | --- | --- | --- | --- | --- | --- | --- | --- | --- | --- | --- | --- | --- | --- | --- | --- | --- | --- | --- | --- | --- | --- | --- | --- | --- | --- | --- | --- | |  | |  |  | | --- | --- | | 15. | Was your region/institution involved in developing the original and/or ongoing collaboration agreement and work plan objectives with NVAP for partnership? \* | |  | Yes No | |  |  |  |  |  | | --- | --- | | 16. | Please assess the implementation and delivery of the NVAP programme to date in your region/institution?\* | |  | |  | | --- | | Significantly exceeded expectations | | Exceeded expectations | | Met expectations | | Required some improvements | | Required significant improvements |  |  | | --- | |  | | |  |  |  |  |  | | --- | --- | |  |  |  |  |  | | --- | --- | | 17. | What have been the main impacts of the NVAP programme for your country/institution? Please select all that apply\* | |  | |  | | --- | | Improved sequencing services | | Streamlining of sequencing workflows | | Improved access to sequencing training and/or expertise | | Upskilling of bioinformatics staff | | Improved use of NGS data for surveillance and/or outbreak response |  Other (please specify) | |  |  |  |  |  | | --- | --- | |  |  |  |  |  | | --- | --- | | 18. | What have been the main benefits of the NVAP programme for your region/institution?\* | |  |  | |  |  |  |  |  | | --- | --- | | 19. | What have been the main limitations of the NVAP programme for your region/insitution?\* | |  |  | |  |  |  |  |  | | --- | --- | | 20. | What additional support would you like the NVAP programme to provide for your region/institution in the future? | |  |  | |  |  |  |  |  | | --- | --- | | 21. | Please highlight **two** examples of NVAP support delivered in your region/institution | |  |  | |  |  |  |  |  | | --- | --- | | 22. | Do you have any other comments or feedback on the NVAP programme? | |  |  | |  |  | |  | | |
