# Supplementary material for: A rapid evaluation of the UK Health Security Agency’s New Variant Assessment Platform global genomic surveillance programme
Source: PLOS Glob Public Health. 2025 Dec 5;5(12):e0005578. doi: 10.1371/journal.pgph.0005578 (PMC12680177; doi:10.1371/journal.pgph.0005578)
Supplement: S3 Survey — (HTML) [file pgph.0005578.s003.html]

 NVAP Informant Survey 


|  |
| --- |
|  |
|  |
| NVAP Informant Survey |

|  |  |  |  |  |  |  |  |  |  |  |  |  |  |  |  |  |  |  |  |  |  |  |  |  |  |  |  |  |  |  |  |  |  |  |  |  |  |  |  |  |  |  |  |  |  |  |  |  |
| --- | --- | --- | --- | --- | --- | --- | --- | --- | --- | --- | --- | --- | --- | --- | --- | --- | --- | --- | --- | --- | --- | --- | --- | --- | --- | --- | --- | --- | --- | --- | --- | --- | --- | --- | --- | --- | --- | --- | --- | --- | --- | --- | --- | --- | --- | --- | --- | --- |
| |  | | --- | |  | | |  |  |  | | --- | --- | --- | |  | | | |  | Background + Section 1 |  | | NVAP Background The New Variant Assessment Platform (NVAP) was launched in April 2021 as part of the COVID-19 pandemic response to support other countries to detect and report new variants of SARS-CoV-2 rapidly. The programme is in its third year of funding and is now focussed on a pathogen agnostic strategy to strengthen genomic surveillance globally. NVAP supports partners in several ways, including genomic sequencing, bioinformatic assessment, characterisation of variants, risk assessment and immunological testing of detected variants of concern (VOCs).   NVAP Evaluation UKHSA’s NVAP team and Evaluation and Epidemiological Science (EES) Division are collaborating on a rapid evaluation of the NVAP programme. This will be the first structured evaluation of the NVAP programme to date and will be completed by the end of October 2023. It is timely to evaluate the NVAP programme to enable contribution to decisions regarding ongoing activity, potential expansion, and future funding beyond March 2024.    NVAP Evaluation Survey This survey will be used to understand your experiences of developing and delivering aspects of the NVAP programme, the lessons learnt and examples of best practice.  Please can you complete this survey by Wednesday 6th September 2023. This survey should take no longer than 20 minutes to complete. If you are unable to complete the survey in a single sitting, your responses will be saved and you will be able to return to the first uncompleted page of the survey when you click on the survey link. You will not be able to amend your answers once the survey has been submitted.   The answers given will be kept in strict confidence and will be held and processed securely in line with the Data Protection Act 2018 and UKHSA information governance policies and procedures. Reporting of the findings will be anonymised so that individual responses cannot be identified.  If you have any queries about this survey or the evaluation, please contact UKHSA’s Evaluation and Epidemiological Science team via email: evaluationepiscience@ukhsa.gov.uk, quoting the reference ‘NVAP Evaluation’ in the email header.   Section 1 |  |  |  |  |  |  |  |  |  |  |  |  |  |  |  |  |  |  |  |  |  |  |  |  |  |  |  |  |  |  |  |  |  |  |  |  |  |  |  |  | | --- | --- | --- | --- | --- | --- | --- | --- | --- | --- | --- | --- | --- | --- | --- | --- | --- | --- | --- | --- | --- | --- | --- | --- | --- | --- | --- | --- | --- | --- | --- | --- | --- | --- | --- | --- | --- | --- | --- | |  | |  |  | | --- | --- | | 1. | Organisation (e.g. UKHSA)\* | |  |  | |  |  |  |  |  | | --- | --- | | 2. | Name of your team (e.g. NVAP team)\* | |  |  | |  |  |  |  |  | | --- | --- | | 3. | Full name of respondent\* | |  |  | |  |  |  |  |  | | --- | --- | | 4. | Job title of respondent\* | |  |  | |  |  |  |  |  | | --- | --- | | 5. | In what capacity have you been involved in the design and delivery of NVAP?\* | |  | |  | | --- | | As an NVAP team member | | As an internal UKHSA NVAP collaborator | | As an external NVAP collaborator working with UKHSA |  |  | | --- | |  | | |  |  |  |  |  | | --- | --- | |  |  | |  | | |

|  |
| --- |
|  |
|  |
| NVAP Informant Survey |

|  |  |  |  |  |  |  |  |  |  |  |  |  |  |  |  |  |  |  |  |  |  |  |  |  |  |  |  |  |  |  |  |  |  |  |  |  |  |  |  |
| --- | --- | --- | --- | --- | --- | --- | --- | --- | --- | --- | --- | --- | --- | --- | --- | --- | --- | --- | --- | --- | --- | --- | --- | --- | --- | --- | --- | --- | --- | --- | --- | --- | --- | --- | --- | --- | --- | --- | --- |
| |  | | --- | |  | | |  |  |  | | --- | --- | --- | |  | | | |  | Section 2: Capability strengthening through technical assistance and training |  | | This section covers your experience working with the NVAP programme in relation to capability strengthening in the form of upskilling staff and providing technical guidance for streamlining the end to end genomic sequencing process. |  |  |  |  |  |  |  |  |  |  |  |  |  |  |  |  |  |  |  |  |  |  |  |  |  |  |  |  |  |  |  | | --- | --- | --- | --- | --- | --- | --- | --- | --- | --- | --- | --- | --- | --- | --- | --- | --- | --- | --- | --- | --- | --- | --- | --- | --- | --- | --- | --- | --- | --- | |  | |  |  | | --- | --- | | 6. | Have you been involved in developing and/or delivering the NVAP offer of capability strengthening through technical assistance and training?\* | |  | Yes No | |  |  |  |  |  | | --- | --- | | 7. | Please select all technical aspects of the NVAP capability strengthening offer that you have been involved in\* | |  | |  | | --- | | Developed the offer (model of workstreams etc.) | | Technical assistance | | Involvement in knowledge exchange sessions with NVAP partners/collaborators | | Development of NVAP training material | | Delivery of NVAP training sessions | | Assistance with External Quality Assessment for SARS-CoV-2 | | Assistance with in-country logistics |  Other (please specify) | |  |  |  |  |  | | --- | --- | |  |  |  |  |  | | --- | --- | | 8. | If possible, please describe any positive experiences or challenges you faced in the development or delivery of NVAP capability strengthening support | |  |  | |  |  | |  | | |

|  |
| --- |
|  |
|  |
| NVAP Informant Survey |

|  |  |  |  |  |  |  |  |  |  |  |  |  |  |  |  |  |  |  |  |  |  |  |  |  |  |  |  |  |  |  |  |  |  |  |  |  |  |
| --- | --- | --- | --- | --- | --- | --- | --- | --- | --- | --- | --- | --- | --- | --- | --- | --- | --- | --- | --- | --- | --- | --- | --- | --- | --- | --- | --- | --- | --- | --- | --- | --- | --- | --- | --- | --- | --- |
| |  | | --- | |  | | |  |  |  | | --- | --- | --- | |  | | | |  | Section 3: Capacity strengthening for pathogen surveillance |  | | This section covers your experience working with the NVAP programme in relation to capacity strengthening in the form of procurement of sequencing infrastructure and assistance/guidance on streamlining sequencing protocols, analysis and reporting to increase sequencing throughput. |  |  |  |  |  |  |  |  |  |  |  |  |  |  |  |  |  |  |  |  |  |  |  |  |  |  |  |  |  | | --- | --- | --- | --- | --- | --- | --- | --- | --- | --- | --- | --- | --- | --- | --- | --- | --- | --- | --- | --- | --- | --- | --- | --- | --- | --- | --- | --- | |  | |  |  | | --- | --- | | 9. | Have you been involved in developing and/or delivering the NVAP offer to support strengthening sequencing capacity in countries/institutions? \* | |  | Yes No | |  |  |  |  |  | | --- | --- | | 10. | Please select all aspects of the offer to increase sequencing volumes that you have been involved in developing and/or delivering as part of the NVAP programme\* | |  | |  | | --- | | Developed the offer (model of workstreams etc.) | | Involved in procurement and/or delivery of sequencing infrastructure | | Involved in registration and/or procurement for External Quality Assessment scheme | | Provided troubleshooting assistance to resolve bottlenecks in sequencing workflows and bioinformatics analysis | | Guidance and training on sequencing workflows and bioinformatic pipelines |  Other (please specify) | |  |  |  |  |  | | --- | --- | |  |  |  |  |  | | --- | --- | | 11. | If possible, please describe any positive experiences or challenges you faced in the development or delivery of the NVAP capacity strengthening support for pathogen surveillance | |  |  | |  |  | |  | | |

|  |
| --- |
|  |
|  |
| NVAP Informant Survey |

|  |  |  |  |  |  |  |  |  |  |  |  |  |  |  |  |  |  |  |  |  |  |  |  |  |  |  |  |  |  |  |  |  |  |  |  |  |  |  |
| --- | --- | --- | --- | --- | --- | --- | --- | --- | --- | --- | --- | --- | --- | --- | --- | --- | --- | --- | --- | --- | --- | --- | --- | --- | --- | --- | --- | --- | --- | --- | --- | --- | --- | --- | --- | --- | --- | --- |
| |  | | --- | |  | | |  |  |  | | --- | --- | --- | |  | | | |  | Section 4: UK Sequencing Support |  | | This section covers your experience working with the NVAP UK sequencing support offer that includes sequencing of samples in the UK and subsequent support for bioinformatic analysis and interpretation |  |  |  |  |  |  |  |  |  |  |  |  |  |  |  |  |  |  |  |  |  |  |  |  |  |  |  |  |  |  | | --- | --- | --- | --- | --- | --- | --- | --- | --- | --- | --- | --- | --- | --- | --- | --- | --- | --- | --- | --- | --- | --- | --- | --- | --- | --- | --- | --- | --- | |  | |  |  | | --- | --- | | 12. | Have you been involved in developing and/or delivering the NVAP offer of UK sequencing support ?\*\* | |  | Yes No | |  |  |  |  |  | | --- | --- | | 13. | Please select all aspects of the NVAP offer of UK Sequencing support that you have been involved in developing and/or delivering\* | |  | |  | | --- | | Provided technical assistance or knowledge exchange with NVAP partners | | Assisted in sample transfer and logistics | | Sequenced samples sent to the UK | | Stored samples | | Shared the sequencing results with partners | | Supported partners with the interpretation of results |  Other (please specify) | |  |  |  |  |  | | --- | --- | |  |  |  |  |  | | --- | --- | | 14. | If possible, please describe any positive experiences or challenges you faced in the development or delivery of the NVAP UK sequencing support | |  |  | |  |  | |  | | |

|  |
| --- |
|  |
|  |
| NVAP Informant Survey |

|  |  |  |  |  |  |  |  |  |  |  |  |  |  |  |  |  |  |  |  |  |  |  |  |  |  |  |  |  |  |  |  |  |  |  |  |  |
| --- | --- | --- | --- | --- | --- | --- | --- | --- | --- | --- | --- | --- | --- | --- | --- | --- | --- | --- | --- | --- | --- | --- | --- | --- | --- | --- | --- | --- | --- | --- | --- | --- | --- | --- | --- | --- |
| |  | | --- | |  | | |  |  |  | | --- | --- | --- | |  | | | |  | Section 5: Biological Risk Assessment for Variants of Interest/Concern |  | | This section covers your experience working with the NVAP programme to receive samples in the UK from international partners in order to provide support for biological risk assessment for further variant characterization analysis or scientific exchange on neutralization study. |  |  |  |  |  |  |  |  |  |  |  |  |  |  |  |  |  |  |  |  |  |  |  |  |  |  |  |  | | --- | --- | --- | --- | --- | --- | --- | --- | --- | --- | --- | --- | --- | --- | --- | --- | --- | --- | --- | --- | --- | --- | --- | --- | --- | --- | --- | |  | |  |  | | --- | --- | | 15. | Have you been involved in developing and/or delivering the NVAP biological risk assessment for variants of interest/concern offer?\* | |  | Yes No | |  |  |  |  |  | | --- | --- | | 16. | Please select all aspects of the NVAP biological risk assessment for variants of interest/concern offer that you have been involved in developing and/or delivering\* | |  | |  | | --- | | Provided technical assistance and knowledge exchange on biological assessment | | Offer to conduct the Biological Risk Assessment testing in UKHSA lab | | Supported NVAP partners in interpretation of the results | | Supported sample transfer and logistics (packaging, sample transport, import permits etc.) |  Other, please specify | |  |  |  |  |  | | --- | --- | |  |  |  |  |  | | --- | --- | | 17. | If possible, please describe any positive experiences or challenges you faced in the development or delivery of the NVAP support for Biological risk assessment for variants of interest/concern offer | |  |  | |  |  | |  | | |

|  |
| --- |
|  |
|  |
| NVAP Informant Survey |

|  |  |  |  |  |  |  |  |  |  |  |  |  |  |  |  |  |  |  |  |  |  |  |  |  |  |  |  |  |  |  |  |  |  |  |  |  |  |  |  |  |  |  |  |  |  |  |  |  |  |  |  |  |  |  |  |  |
| --- | --- | --- | --- | --- | --- | --- | --- | --- | --- | --- | --- | --- | --- | --- | --- | --- | --- | --- | --- | --- | --- | --- | --- | --- | --- | --- | --- | --- | --- | --- | --- | --- | --- | --- | --- | --- | --- | --- | --- | --- | --- | --- | --- | --- | --- | --- | --- | --- | --- | --- | --- | --- | --- | --- | --- | --- |
| |  | | --- | |  | | |  |  |  | | --- | --- | --- | |  | | | |  | Section 6: NVAP Views and Experiences |  | | This section covers your overall experience working with the NVAP programme to date |  |  |  |  |  |  |  |  |  |  |  |  |  |  |  |  |  |  |  |  |  |  |  |  |  |  |  |  |  |  |  |  |  |  |  |  |  |  |  |  |  |  |  |  |  |  |  |  | | --- | --- | --- | --- | --- | --- | --- | --- | --- | --- | --- | --- | --- | --- | --- | --- | --- | --- | --- | --- | --- | --- | --- | --- | --- | --- | --- | --- | --- | --- | --- | --- | --- | --- | --- | --- | --- | --- | --- | --- | --- | --- | --- | --- | --- | --- | --- | |  | |  |  | | --- | --- | | 18. | Have you been involved in the development and/or delivery of any aspect of the NVAP programme to date?\* | |  | Yes No | |  |  |  |  |  | | --- | --- | | 19. | How would you assess your experience in developing and/or delivering the NVAP programme?\* | |  | |  | | --- | | 1. Significantly exceeded expectations | | 2. Exceeded expectations | | 3. Met expectations | | 4. Required some improvements | | 5. Required significant improvements |  |  | | --- | |  | | |  |  |  |  |  | | --- | --- | |  |  |  |  |  | | --- | --- | | 20. | Please highlight two examples of the impact of NVAP in strengthening global genomic surveillance relevant to your area of involvement in NVAP\* | |  |  | |  |  |  |  |  | | --- | --- | | 21. | Please describe any enablers that you faced in the overall development and/or delivery of the NVAP programme to date\* | |  |  | |  |  |  |  |  | | --- | --- | | 22. | Please describe any limitations that you faced in the overall development and/or delivery of the NVAP programme to date\* | |  |  | |  |  |  |  |  | | --- | --- | | 23. | Do you have any other feedback on the NVAP programme, or recommendations for the future? | |  |  | |  |  | |  | | |
